# Adequacy of the Risk Stratification Instrument in Mental Health: Experience Report

**DOI:** 10.1590/0034-7167-2025-0037

**Published:** 2025-12-08

**Authors:** Mariana Cláudio da Silva Sartori Nakamura, Guilherme Correa Barbosa, Rúbia Aguiar Alencar

**Affiliations:** I Universidade Estadual Paulista. Botucatu, São Paulo, Brazil

**Keywords:** Mental Health, Primary Health Care, Risk Assessment, Risk Classification, Multidisciplinary Team., Salud Mental, Atención Primaria de Salud, Medición del Riesgo, Clasificación del Riesgo, Equipo Multiprofesional.

## Abstract

**Objectives:**

to report the process of adapting a Mental Health Risk Stratification Instrument through a workshop.

**Methods:**

experience report of the process of adapting an instrument, which is not validated, through a workshop with health professionals from the Psychosocial Care Network of a medium-sized city, using Paulo Freire’s dialogical pedagogy as a methodological framework.

**Results:**

the workshop enabled dialogue, reflection and collective construction. The professionals identified weaknesses in the instrument, such as unclear descriptors, lack of criteria that considered socio-family and subjective aspects, and doubts regarding the applicability of risk categories. They suggested adjustments, discussed disagreements and sought consensus.

**Final Considerations:**

mutual listening and the appreciation of professional experiences favored the appropriation of the instrument by the participants, strengthening the commitment to the qualification of mental health care and positively impacting the improvement of the work processes of the teams.

## RESUMEN

**Objetivos:**

informar sobre el proceso de adaptación de un Instrumento de Estratificación del Riesgo en Salud Mental.

**Métodos:**

informe de experiencia del proceso de adaptación de un instrumento no validado, mediante un taller con profesionales sanitarios de la Red de Atención Psicosocial de un municipio de medio porte, utilizando la pedagogía dialógica de Paulo Freire como referencia metodológica.

**Resultados:**

el taller permitió diálogos, reflexiones y una construcción colectiva. Los profesionales identificaron puntos débiles en la herramienta, como descriptores poco claros, falta de criterios que ponderasen aspectos sociofamiliares y subjetivos, y dudas sobre la aplicabilidad de las categorías de riesgo. Propusieron ajustes, debatieron diferencias y buscaron consenso.

**Consideraciones Finales:**

la escucha mutua y la valorización de las experiencias profesionales favorecieron la apropiación del instrumento, fortaleciendo el compromiso con la cualificación de la atención en salud mental e impactando positivamente en la mejora de los procesos de trabajo de los equipos.

## INTRODUCTION

In this study, mental health is conceptualized as a state of subjective, relational, and socially constructed well-being in which the individual recognizes his or her capabilities, faces the challenges of daily life, and meaningfully participates in his or her community. It is a complex notion, influenced by social, economic, cultural, and affective determinants, which cannot be simply reduced to the absence of mental disorders^(1)^.

The complexity of mental health is highlighted, which involves recognizing psychological distress and the links in the territories. In this scenario, it is essential to have instruments that support health professionals, guide care, and organize demand. Risk Stratification in Mental Health emerges as a strategic tool for clinical and managerial decisions in Primary Health Care (PHC), strengthening comprehensive care and coordination with the Psychosocial Care Network (PSCN)^(1,2)^.

In Brazil, PHC is the main gateway to the SUS and plays a central role in coordinating care, including in mental health. However, it faces challenges such as insufficient training of professionals, fragmentation of actions, disarticulation of the work process and difficulties in implementing comprehensive care practices in the face of complex demands^(3)^.

In this context, Mental Health Risk Stratification enables a classification of the risk of mental illness based on severity, social vulnerability and available care resources, contributing to the most appropriate allocation of team efforts, favoring the health work process^(2,3)^. This tool assists not only in planning actions, but also for optimizing care flows and strengthening a logic of care centered on the subject^(2,4)^.

Despite the potential of the available instruments to qualify care and support the work process of health teams, their effective implementation still faces obstacles such as lack of time, work overload of teams, lack of specific training, precarious working conditions, insufficient funding, professional training misaligned with the care model centered on PHC, fragile employment relationships with institutions and difficulties in intersectoral coordination^(5)^. Such factors compromise the consolidation of comprehensive care practices, in line with the principles of the Family Health Strategy (FHU), which have been progressively strained by the predominance of the biomedical model in recent years^(1,5)^.

In light of this scenario, it is essential to invest in ongoing education, raising awareness among teams, and creating instruments that address local specificities, value community ties, and encourage emancipatory practices^(5)^. The use of instruments such as the Mental Health Risk Stratification Instrument should be seen as part of a broader process of qualifying care and monitoring the health work process, with systematic evaluation of results^(4,5)^.

Therefore, the Mental Health Risk Stratification Instrument is a strategic tool in the work process of PHC teams, as long as it is inserted in a context that involves institutional support, ongoing training of professionals, expanded listening, and a commitment to comprehensive care.

However, there is a knowledge gap regarding the practical applicability and suitability of these instruments to the concrete realities of the services, which justifies this study, as our purpose is to adapt a Mental Health Risk Stratification Instrument through a workshop with health professionals from the PSCN of a medium-sized municipality. The research is based on the premise that it is necessary to qualify the work process of PSCN professionals, since the use of these instruments contributes to the planning, reception and management of care, respecting the uniqueness of individuals and territories.

## OBJECTIVES

To report the process of adapting a Mental Health Risk Stratification Instrument through a workshop.

## METHODS

This is an experience report developed in a medium-sized city located in the state of São Paulo, Brazil, with an estimated population of 145,000 inhabitants. The city has two School Health Centers linked to a Public College, which operate as a traditional model with multidisciplinary therapeutic offerings linked to education. There are also six PHC units, 13 Family Health Units (FHU) with 21 teams, a Street Clinic and two eMulti teams, covering approximately 95% of the city’s population. There is also a polyclinic (with outpatient clinics for transgender, acupuncture, homeopathy and physiotherapy), an occupational health service, and two Psychosocial Care Centers (CAPS), one CAPS I and one CAPSi II.

This report describes the experience of adapting a Mental Health Risk Stratification Instrument, carried out during a workshop divided into two meetings. The activity adopted Paulo Freire’s dialogical pedagogy as a methodological framework, based on dialogue, critical thinking, and the collective construction of knowledge^(6)^. The objective was to review and improve the instrument and its descriptors with the participation of professionals who would use it in the future in the daily lives of Primary Health Care (PHC) teams.

The Mental Health Risk Stratification Instrument, developed by the Mental Health Division of the Paraná State Health Department (SESA), has not been validated yet. It is based on the main mental disorders of the WHO, organized according to the criteria of the International Classification of Diseases (ICD-10) and the Diagnostic and Statistical Manual of Mental Disorders (DSM-5-R)^(2)^, including symptoms, aggravating and mitigating factors. In 2020, it underwent a review with the support of the Council of Municipal Health Departments of Paraná, Regional Health Departments, and municipalities, aiming to qualify shared care between PHC and specialized care. The tool contributes to the planning and organization of mental health care in the territory^(4)^.

After the researcher contacted SESA, authorization was granted to use the instrument, with the purpose of adapting it to the reality of the municipality under study. It is important to note that scientific validation with expert judges will be carried out in future research.

The instrument is composed of five groups: 1. Symptoms related to common or minor mental disorders; 2. Symptoms related to severe and persistent mental disorders; 3. Symptoms related to dependence on alcohol and other drugs; 4. Symptoms related to changes in mental health that manifest in childhood and/or adolescence; 5. Factors that may constitute aggravating or mitigating factors of previously identified mental health problems, including special conditions and acute events.4 Each group is composed of items, totaling 54 items in the end. In addition to the instrument, there is another document composed of the descriptors of each item.

All higher education professionals from the PSCN, under municipal management, were invited, prioritizing those from the APS, including social workers, dentists, nurses, physiotherapists, doctors, nutritionists, physical education professionals, psychologists, and municipal service managers, totaling approximately 85 professionals.

The invitations were sent via WhatsApp and email to the employees mentioned above, through the municipality’s Health Education and Communication Sector. The invitation included a registration link linked to the Technical Section for Support to Teaching, Research, and Extension of the Public College, which offered the possibility of choosing between the morning or afternoon period, considering the need to maintain care in the RAS, without compromising services.

The meetings took place in the mornings of September and October 2022, respectively, and were repeated in the afternoons, with the intention of providing the opportunity for more professionals to participate while maintaining activities in the health services. There were 45 professionals present at the first meeting and 33 at the second. The study sample was considered to be 33 professionals, as they were the people who attended both meetings of the First Workshop. Failure to participate in both workshops constituted an exclusion criterion.

The objectives of the First Meeting of the workshop were to present the object of study, learn about the reality of the participants, and begin the adaptation of the Mental Health Risk Stratification Instrument. At the time, ethical precepts were followed, a participation contract was signed, and materials were provided, including the Logbook with the instrument, descriptors, Free and Informed Consent Form, sheets for notes and evaluation.

The participants were divided into two groups and invited to reflect on the question “For you, what is mental health?”. They then analyzed the instrument and proposed improvements. Each group elected a secretary (to record and control time) and a rapporteur (to present to the plenary). The suggestions were discussed and incorporated in real time, using multimedia resources, allowing collective consensus. At the end, an evaluation of the meeting was made.

The Second Meeting began with the presentation of the summary of the triggering question and the results of the previous evaluation, through a word cloud generated by Wordle. The groups resumed work, continuing to adapt the instrument. After completion, suggestions were presented, resulting in an instrument adjusted to the local reality. The meeting ended with a new evaluation.

The research was approved by the Research Ethics Committee on March 7^th^, 2022, opinion No. 5,278,279, CAAE:55988922.0.0000.5411.

## RESULTS

The workshop held with professionals from the PSCN proved to be a fruitful space for dialogue, reflection and collective construction. More than a training moment, the workshop was configured as a live exercise in articulating theory and practice, bringing technical knowledge closer to the concrete reality of the territory where mental health care is provided.

The workshop was attended by 33 professionals, distributed as follows: three social workers (9.1%), two dentists (6.0%), 16 nurses (48.5%), one pharmacist (3.0%), four doctors (12.1%) and seven psychologists (21.3%). The majority of participants were female, with ages ranging from 25 to 58 years old, with an average age of 38 years old. The diversity of backgrounds, professional experiences and life trajectories contributed significantly to enriching the discussions held during the workshop.

During the First Meeting, based on the triggering question “What is mental health for you?”, multiple perceptions emerged, with words such as balance, listening, suffering, relief, care and quality of life standing out in the responses. The construction of a word cloud allowed us to visualize the plurality of meanings attributed to the topic, reflecting the expanded view that PSCN professionals have been constructing in the daily life of the SUS.

The analysis of the original Mental Health Risk Stratification instrument was carried out in small groups, allowing for greater depth and participation. The professionals’ criticism was based not only on technical references, but, above all, on their practical experiences. Important weaknesses were identified, such as unclear descriptors, the absence of criteria that considered the socio-family and subjective aspects of users, and doubts regarding the applicability of certain risk categories.

The groups demonstrated great engagement, proposing consistent and contextualized reformulations. However, the final analysis of the instrument took place at the Second Meeting.

At the end of the First Meeting, an evaluation was carried out with open questions: “What did I learn?”, “What did I build?”, “What did I feel?”. The answers revealed the participants’ perception that they were not just reviewing an instrument, but participating in a process of professional appreciation, exchange of knowledge and reconstruction of care. They highlighted the feeling of belonging, the importance of the listening space and the recognition of their experiences as fundamental for the construction of more qualified care.

The Second Meeting revisited the main points discussed previously and advanced the analysis of the instrument. Through in-depth discussions, significant changes were proposed, including the addition of item 55, and the other items were reformulated in their wording, considering the needs pointed out by the participants.

In addition to Table 1, there is the material with the descriptors of each item of the Risk Stratification instrument. These descriptors were also evaluated by the participants and underwent modifications.

During the plenary session, the two groups presented their suggestions for adjustments, discussed points of divergence and sought to build consensus. All modifications and additions are systematized in Table 1 (readjusted instrument), with highlights in bold. This approach allowed the alignment of the different knowledge brought by the participants around a common theme, promoting a deeper understanding and favoring reflection on the practice. The following questions were asked to evaluate the second meeting: “How do I feel having participated in this construction?”, “What contributions from the workshop will I take to work?”, “What are my expectations regarding the risk stratification instrument for my professional practice?”. The main words mentioned in the final evaluation of the Second Meeting were: opportunity, exchange of experience, training, qualifying, assistance, comprehensive approach and welcoming, which summarize the formative impact of the activity, both in the technical improvement of the instrument and in the strengthening of the bonds between the professionals and their daily practices. This fact favors the improvement of the work process of these health professionals.

During the meetings, the group’s growth in terms of ownership of the process and commitment to improving mental health care was observed. Mutual listening, respect for different experiences, and the collective desire to build a tool that is more sensitive to local reality contributed to the workshop becoming a significant experience in continuing health education.

In short, the process resulted not only in the construction of an instrument that is appropriate to the reality of the municipality, but also in improving the work process of health teams, consequently contributing to improving care for people with mental illness.

The choice of Paulo Freire’s dialogical pedagogy as the basis for organizing the workshop highlighted the value of participatory practices as a strategy to strengthen professional bonds, promote co-accountability, and improve mental health care.

## DISCUSSION

The workshop, organized based on Paulo Freire’s dialogical pedagogy, enabled the adaptation of a Mental Health Risk Stratification Instrument to the reality of a medium-sized city. This collaborative experience among the PSCN professionals who participated in the workshop favored the identification of gaps in the original instrument and allowed the insertion and/or modification of clearer criteria for the daily routine of services and the specificities of the population served, as recommended by the literature on health assessment technologies^(6,7)^.

**Chart 1:** Appropriate Mental Health Risk Stratification Instrument sent for judges’ evaluation

RISK STRATIFICATION IN MENTAL HEALTH						
**Name:**		Medical Record Number:				
DN:		Age:				
Sexual Orientation:		Education:				
Occupation:		Race/color:				
Marital status:						
Name and professional registration:						
Health service:			Date:			
**SIGNS AND SYMPTOMS**					**NO**	**YES**
GROUP I (Symptoms related to common or minor mental disorders)	1. Anxiety with or without feeling of panic				0	4
	2. Insomnia or hypersomnia				0	3
	3. Phobia (intense fear of something without real risk)				0	2
	4. Conversion crises				0	2
	5. Dissociative seizures				0	2
	**6.** Changes in appetite and **eating behavior**				0	2
	7. Excessive concern about weight or body shape				0	2
	**8. Hypochondriacal symptoms** and/or physical complaints **without organic cause**				0	2
	**9. Obsessive thoughts and/or compulsive behaviors**				0	2
	10. Thoughts of worthlessness and/or feelings of guilt				0	4
	11. Persistent sadness with loss of interest and pleasure and/or hopelessness				0	4
	12. Apathy with or without social isolation				0	4
	13. Impairment of sexual activity				0	2
	14. Temporal and/or spatial disorientation				0	2
GROUP II (Symptoms related to severe and persistent mental disorders)	**15. Passive thoughts of death**				0	2
	**16. Suicidal ideation without planning**				0	4
	**17. Suicidal ideation with planning or recent suicide attempt.**				0	10
	18. Unstable mood with impulsivity or destructiveness				0	6
	19. Heteroaggressiveness or self-aggressiveness				0	8
	20. Social or sexual disinhibition or loss of modesty.				0	4
	21. Motor hyperactivity				0	4
	22. Elevated, expansive, irritable, or euphoric mood				0	4
	23. Delirium				0	8
	24. Alucinação				0	8
	25. Change in the course and/or form of thought				0	6
	26. Loss of critical capacity of reality				0	8
	27. Memory alteration				0	2
GROUP III (Symptoms related to alcohol and other drug dependence)	*28. Delirium tremen**s***				0	10
	29. Signs or symptoms of withdrawal from continued use of alcohol and/or drugs				0	8
	30. Inability to reduce and control drug use				0	8
	31. Risky behavior, for oneself or others, under the influence of alcohol or drugs				0	8
	32. Tolerance to the effects of alcohol consumption or legal and illegal drugs				0	6
	33. **Harmful** use of alcohol or drugs				0	8
GROUP IV (Symptoms related to changes in mental health that manifest in childhood and/or adolescence)	34. Difficulty understanding and/or transmitting verbal information manifested during the period of child development				0	4
	35. Repetitive, atypical, or paralyzed body or behavioral movements.				0	4
	36. Difficulty acquiring and developing school skills				0	4
	37. Difficulty acquiring and developing motor skills				0	4
	38. Severe difficulty in social interaction and changes in routine				0	8
	39. Inattention and/**or hyperactivity** with premature interruption of tasks and/or leaving tasks unfinished				0	2
	40. Persistent provocative, defiant and/or oppositional behavior				0	6
	41. Behaviors or emotional reactions that do not correspond to what is expected for biological age				0	4
GROUP V (Factors that may constitute aggravating or mitigating factors for already identified mental health problems, including special conditions and acute events)	42. Resistance, refractoriness or non-adherence to treatment.				0	4
	43. Recurrence or **Relapse** (after 2 months of **stabilization**)				0	4
	44. Continued exposure to stress or traumatic event				0	4
	45. Precarious family and/or social support				0	4
	46. Witness to violence				0	2
	47. Perpetrator or victim of interpersonal violence				0	6
	48. Loss of autonomy				0	6
	49. **Loss of functional/occupational capacity** resulting from health problems				0	4
	50. Social Vulnerability				0	2
	51. Family history of mental disorder/chemical dependency/suicide				0	2
	52. Comorbidity or other chronic health condition				0	4
	53. Age range < 18 years and > 60 years				0	2
	54. School dropout and/or delay				0	2
	**55. Polypharmacy**				0	2
SCORE	RISK					
0 a 42	LOW RISK					
43 a 71	MÉDIUM RISK					
72 a 244	HIGH RISK					
TOTAL SCORE:						

More than a participatory strategy, the workshop is essential to stimulate the exchange of knowledge, train and drive the continuous improvement of professionals, qualifying mental health care. It favors a collaborative approach, promoting learning, exchange of experiences and building skills among professionals from different areas. Furthermore, it creates an environment for sharing experiences, discussing cases and critically analyzing the flow of care, adjusting the risk criteria to the observed realities^(8)^.

One of the main results observed was the redefinition of the Mental Health Risk Stratification Instrument by the study participants themselves. By analyzing the criteria initially proposed, the participants were able to propose adjustments and create new categories, based on their daily experience. This movement dialogues with the idea that technical instruments must be contextualized, legitimized by the collective and capable of reflecting the reality of those who apply them^(9)^.

Although the professional diversity of the group imposed challenges regarding the alignment of perceptions, it also enriched the process, as it allowed multiple perspectives on mental health risk. The literature has emphasized that the interprofessional construction of assessment instruments increases their legitimacy and enhances their use in daily life^(10)^.

In addition, the participatory construction of the instrument contributed to the appropriation of its purpose by professionals, which tends to favor its practical application and improve the work process of health teams. Understanding the “why” and “what for” using an instrument is a determining factor for its effectiveness in services, as indicated by studies on the implementation of health technologies^(5,7)^.

The adopted strategy allowed for reflection on the flow of mental health care in the PSCN, promoting critical analysis of routines and encouraging innovative practices. The sense of belonging generated in the meetings strengthened the participants’ commitment to adapting the instrument, demonstrating that participatory processes are essential for creating more effective technologies, contextually validated and oriented towards comprehensive care^(9,10)^.

Finally, the active participation of PSCN professionals in a dialogical and democratic process for adapting the Mental Health Risk Stratification Instrument is a strategy with the potential to qualify mental health care in the PHC, in a critical and contextualized manner, promoting improvements in the work process of health teams.

### Study limitations

The study faced limitations such as the low adherence of municipal managers to the adaptation of the Mental Health Risk Stratification instrument, which could favor its future implementation. In addition, the participatory approach adopted, enabling intense subjective exchange among participants, may not have fully captured collective expressions and perceptions, although the data obtained were sufficient to support the analyses and reflect the lived experience.

### Contributions to the Area

By adopting Freirean pedagogy, the study promotes an emancipatory approach, strengthening critical practices in health and qualifying the work process of teams, in line with the principles of the SUS. It contributes to Nursing by proposing improvements in care, encouraging co-responsibility in mental health and valuing the leadership of teams in the creation of technologies appropriate to the territories.

## FINAL CONSIDERATIONS

This experience report highlighted the workshop’s potential as a space for training, listening and collective reconstruction of mental health care in PHC. The adaptation of the Mental Health Risk Stratification Instrument, through the active participation of PSCN professionals, proved to be not just a technical exercise of reviewing items, but a living pedagogical practice that connected knowledge, realities and feelings.

The contributions of participants, from different professional categories, improved the stratification criteria and expanded the understanding of psychological distress. At the end of the process, the participants not only modified the instrument, but also recognized themselves as active subjects in the construction of more qualified, humane and territorialized care.

The revision of the instrument, based on practical experience, refined the criteria and increased its clarity, strengthening its role in supporting clinical decision-making, the organization of mental health care and, consequently, in improving the work process of health teams.

## Data Availability

The research data are available only upon request.
