# Supplementary material for: Adequacy of the Risk Stratification Instrument in Mental Health: Experience Report
Source: Rev Bras Enferm. 2025 Dec 8;78(6):e20250037. doi: 10.1590/0034-7167-2025-0037 (PMC12707614; doi:10.1590/0034-7167-2025-0037)
Supplement: Translation

# Translation: Adequação do Instrumento de Estratificação de Risco em Saúde Mental: Relato de Experiência

**DOI:** 10.1590/0034-7167-2025-0037pt

**Authors:** Mariana Cláudio da Silva Sartori Nakamura, Guilherme Correa Barbosa, Rúbia Aguiar Alencar

**Affiliations:** I Universidade Estadual Paulista. Botucatu, São Paulo, Brasil

**Keywords:** Saúde Mental, Atenção Primária à, Saúde, Medição de Risco, Classificação de Risco, Equipe multiprofissional.

## Abstract

**Objetivos:**

relatar o processo de adequação de um Instrumento de Estratificação de Risco em Saúde Mental por meio de uma oficina.

**Métodos:**

relato de experiência do processo de adequação de um instrumento, que não é validado, por meio de oficina com profissionais de saúde da Rede de Atenção Psicossocial de um município de médio porte, utilizando como referencial metodológico a pedagogia dialógica de Paulo Freire.

**Resultados:**

a oficina possibilitou o diálogo, reflexão e construção coletiva. Os profissionais identificaram fragilidades no instrumento, como descritores pouco claros, ausência de critérios que considerassem aspectos sociofamiliares e subjetivos, e dúvidas quanto à aplicabilidade de categorias de risco. Sugeriram ajustes, debateram divergências e buscaram consensos.

**Considerações Finais:**

a escuta mútua e a valorização das experiências profissionais favoreceram a apropriação do instrumento pelos participantes, fortalecendo o compromisso com a qualificação do cuidado em saúde mental e impactando positivamente na melhoria dos processos de trabalho das equipes.

## INTRODUÇÃO

Neste estudo, a saúde mental é conceituada como um estado de bem-estar subjetivo, relacional e socialmente construído, no qual o indivíduo reconhece suas capacidades, enfrenta os desafios da vida cotidiana e participa de maneira significativa em sua comunidade. Trata-se de uma noção complexa, influenciada por determinantes sociais, econômicos, culturais e afetivos, que não pode ser simplesmente reduzida à ausência de transtornos mentais^(1)^.

Destaca-se a complexidade da saúde mental, que envolve reconhecer o sofrimento psíquico e os vínculos nos territórios. Nesse cenário, é essencial dispor de ferramentas que apoiem os profissionais de saúde, orientem o cuidado e organizem a demanda. A Estratificação de Risco em Saúde Mental surge como uma ferramenta estratégica para as decisões clínicas e gerenciais na Atenção Primária à Saúde (APS), fortalecendo o cuidado integral e a articulação com a Rede de Atenção Psicossocial (RAPS)^(1,2)^.

No Brasil, a APS é a principal porta de entrada do SUS e desempenha um papel central na coordenação do cuidado, inclusive em saúde mental. No entanto, enfrenta-se desafios como a formação insuficiente dos profissionais, fragmentação das ações, desarticulação do processo de trabalho e dificuldades para efetivar práticas de cuidado integral diante de demandas complexas^(3)^.

Nesse contexto, a Estratificação de Risco em Saúde Mental, possibilita uma **classificação do risco do adoecimento mental** com base na gravidade, na vulnerabilidade social e nos recursos de cuidado disponíveis, contribuindo para a alocação mais adequada de esforços da equipe, favorecendo o processo de trabalho em saúde^(2,3)^. Essa ferramenta auxilia não apenas no planejamento de ações, mas também na otimização dos fluxos assistenciais e no fortalecimento de uma lógica de cuidado centrada no sujeito^(2,4)^.

Apesar do potencial das ferramentas disponíveis para qualificar o cuidado e apoiar o processo de trabalho das equipes de saúde, sua efetiva implementação ainda enfrenta obstáculos como a escassez de tempo, a sobrecarga de trabalho das equipes, a falta de capacitação específica, as condições precárias de trabalho, o financiamento insuficiente, à formação profissional desalinhada com o modelo de atenção centrado na APS, a fragilidade nos vínculos empregatícios com as instituições e as dificuldades na articulação intersetorial^(5)^. Tais fatores comprometem a consolidação de práticas de cuidado integral, em consonância com os princípios da Estratégia Saúde da Família (ESF), que têm sido progressivamente tensionados pela predominância do modelo biomédico nos últimos anos^(1,5)^.

Diante desse cenário, é essencial investir na educação permanente, sensibilização das equipes e na criação de instrumentos que dialoguem com as especificidades locais, valorizem os vínculos comunitários e estimulem práticas emancipatórias^(5)^. A utilização de ferramentas como o Instrumento de Estratificação de Risco em Saúde Mental deve ser vista como parte de um processo mais amplo de qualificação do cuidado e monitoramento do processo de trabalho em saúde, com avaliação sistemática dos resultados^(4,5)^.

Assim, compreende-se que o Instrumento de Estratificação de Risco em Saúde Mental é uma ferramenta estratégica no processo de trabalho das equipes da APS, desde que esteja inserida em um contexto que envolva apoio institucional, formação contínua dos profissionais, escuta ampliada e um compromisso com o cuidado integral.

Contudo, existe uma lacuna de conhecimento sobre a aplicabilidade prática e a adequação dessas ferramentas às realidades concretas dos serviços, o que justifica a realização deste estudo, que visou adequar um Instrumento de Estratificação de Risco em Saúde Mental por meio de uma oficina com profissionais de saúde da RAPS de um município de médio porte. A pesquisa parte da premissa de que é necessário qualificar o processo de trabalho dos profissionais da RAPS, uma vez que, a utilização dessas ferramentas contribui para o planejamento, acolhimento e gestão do cuidado, respeitando a singularidade dos indivíduos e dos territórios.

## OBJETIVOS

Relatar o processo de adequação de um Instrumento de Estratificação de Risco em Saúde Mental por meio de uma oficina.

## MÉTODOS

Trata-se de um relato de experiência desenvolvido em um município de médio porte, localizado no interior do estado de São Paulo, Brasil, com população estimada de 145.000 habitantes. A cidade conta com dois Centros de Saúde Escola vinculados a uma Faculdade Pública, os quais funcionam como modelo tradicional com ofertas terapêuticas multiprofissionais ligadas ao ensino. Há também seis unidades de APS, 13 unidades de Saúde da Família (ESF) com 21 equipes, um Consultório de Rua e duas equipes eMulti, cobrindo cerca de 95% da população do município. Há também uma policlínica (com ambulatórios transexualizador, de acupuntura, homeopatia e fisioterapia), um serviço de saúde do trabalhador, e dois Centros de Atenção Psicossocial (CAPS), sendo um CAPS I e um CAPSi II.

Este relato descreve a experiência de adequação de um Instrumento de Estratificação de Risco em Saúde Mental, realizada durante uma oficina dividida em dois encontros. A atividade adotou como referencial metodológico a pedagogia dialógica de Paulo Freire, pautada no diálogo, na criticidade e na construção coletiva do saber^(6)^. O objetivo foi revisar e aprimorar o instrumento e seus descritores com a participação dos profissionais que, futuramente, o utilizariam no cotidiano das equipes da Atenção Primária à Saúde (APS).

O Instrumento para Estratificação de Risco em Saúde Mental, desenvolvido pela Divisão de Saúde Mental da Secretaria de Estado da Saúde do Paraná (SESA), ainda não foi validado. Baseia-se nos principais transtornos mentais da OMS, organizados conforme os critérios da Classificação Internacional de Doenças (CID-10) e do Manual Diagnóstico e Estatístico de Transtornos Mentais (DSM-5-R)^(2)^, incluindo sintomas, fatores agravantes e atenuantes. Em 2020, o referido instrumento passou por revisão com apoio do Conselho de Secretarias Municipais de Saúde do Paraná, Regionais de Saúde e municípios, visando qualificar o cuidado compartilhado entre a APS e a especializada. A ferramenta contribui para o planejamento e organização da atenção em saúde mental no território^(4)^.

Após contato da pesquisadora com a SESA, foi concedida autorização para utilização do instrumento, com o propósito de adequá-lo à realidade do município do estudo. Ressalta-se que a validação cientifica junto a juízes especialistas será realizada em pesquisa futura.

O instrumento é composto por cinco grupos:1. Sintomas relacionados aos transtornos mentais comuns ou menores; 2. Sintomas relacionados aos transtornos mentais severos e persistentes; 3. Sintomas relacionados à dependência de álcool e outras drogas; 4. Sintomas relacionados a alterações na saúde mental que se manifestam na infância e/ou na adolescência; 5. Fatores que podem se constituir em agravantes ou atenuantes de problemas de saúde mental já identificado, incluindo condições especiais e eventos agudos^(4)^. Cada grupo é composto por itens, totalizando 54 itens. Além do instrumento, há outro documento composto pelos descritores de cada item.

Foram convidados todos os profissionais de nível superior da RAPS, sob gestão municipal, priorizando os da APS, dentre eles, assistentes sociais, cirurgiões-dentistas, enfermeiros, fisioterapeutas, médicos, nutricionistas, profissionais de educação física, psicólogos, além dos gestores municipais dos serviços, totalizando aproximadamente 85 profissionais.

Os convites foram enviados via *WhatsApp* e por e-mail para os colaboradores citados acima, por meio do Setor de Educação e Comunicação em Saúde do município. No convite, havia o *link* para inscrição vinculado à Seção Técnica de Apoio ao Ensino, Pesquisa e Extensão da Faculdade Pública, que ofereceu a possibilidade de escolha entre o período da manhã ou tarde, considerando a necessidade de manter o cuidado na RAS, sem prejuízos aos serviços.

Os encontros ocorreram, respectivamente, nos meses de setembro e outubro de 2022, no período da manhã e foram repetidos no período da tarde com a intenção de oportunizar a participação de mais profissionais e, ao mesmo tempo, manter as atividades nos serviços de saúde. Estiveram presentes 45 profissionais no primeiro e 33 no segundo encontro. Para a amostra do estudo, considerou-se o quantitativo de 33 profissionais, pois foram as pessoas que estiveram presentes nos dois encontros da Primeira Oficina. A não participação nas duas oficinas configurou critério de exclusão.

O Primeiro Encontro da oficina teve como objetivos: apresentar o objeto de estudo, conhecer a realidade dos participantes e iniciar a adaptação do Instrumento de Estratificação de Risco da Saúde Mental. Na ocasião, foram seguidos os preceitos éticos, firmado o contrato de participação e entregues os materiais, incluindo o Diário de Bordo com o instrumento, descritores, Termo de Consentimento Livre e Esclarecido, folhas para anotações e avaliação.

Os participantes foram divididos em dois grupos e convidados a refletir sobre a pergunta “Para você, o que é saúde mental?”. Em seguida, analisaram o instrumento e propuseram melhorias. Cada grupo elegeu um secretário (para registrar e controlar o tempo) e um relator (para apresentar à plenária). As sugestões foram discutidas e incorporadas em tempo real, com uso de recursos multimídia, permitindo o consenso coletivo. Ao final, foi feita uma avaliação do encontro.

O Segundo Encontro iniciou com a apresentação da síntese da pergunta disparadora e dos resultados da avaliação anterior, por meio de uma nuvem de palavras gerada pelo Wordle. Os grupos retomaram os trabalhos, continuando as adequações do instrumento. Após a finalização, as sugestões foram apresentadas, resultando em um instrumento ajustado à realidade local. O encontro foi encerrado com nova avaliação.

A pesquisa foi aprovada pelo Comitê de Ética em Pesquisa no dia 07 de março de 2022, parecer n° 5.278.279, CAAE:55988922.0.0000.5411.

## RESULTADOS

A oficina realizada com profissionais da RAPS revelou-se um espaço fecundo de diálogo, reflexão e construção coletiva. Mais do que um momento formativo, a oficina configurou-se como um exercício vivo de articulação entre teoria e prática, aproximando o saber técnico da realidade concreta do território onde se faz o cuidado em saúde mental.

Participaram da oficina 33 profissionais, distribuídos da seguinte forma: três assistentes sociais (9,1%), dois cirurgiões-dentistas (6,0%), 16 enfermeiros (48,5%), um farmacêutico (3,0%), quatro médicos (12,1%) e sete psicólogos (21,3%). A maioria dos participantes era do sexo feminino, com idades variando entre 25 e 58 anos, e média etária de 38 anos. A diversidade de formações, experiências profissionais e trajetórias de vida contribuiu significativamente para o enriquecimento das discussões realizadas durante a oficina

No Primeiro Encontro, a partir da pergunta disparadora “Para você, o que é saúde mental?”, emergiram múltiplas percepções, destacando-se, nas respostas, palavras como equilíbrio, escuta, sofrimento, alívio, cuidado e qualidade de vida. A construção de uma nuvem de palavras permitiu visualizar a pluralidade de sentidos atribuídos ao tema, refletindo o olhar ampliado que os profissionais da RAPS vêm construindo no cotidiano do SUS.

A análise do instrumento original de Estratificação de Risco em Saúde Mental foi realizada em pequenos grupos, permitindo maior profundidade e participação. A crítica dos profissionais se fundamentou não apenas em referenciais técnicos, mas, sobretudo, em suas vivências práticas. Identificou-se fragilidades importantes, como descritores pouco claros, ausência de critérios que considerassem aspectos sociofamiliares e subjetivos dos usuários, e dúvidas quanto à aplicabilidade de certas categorias de risco.

Os grupos demonstraram grande engajamento, propondo reformulações consistentes e contextualizadas. No entanto, a análise final do instrumento aconteceu no Segundo Encontro.

Ao final do Primeiro Encontro, uma avaliação foi realizada com perguntas abertas: “O que conheci?”, “O que construí?”, “O que senti?”. As respostas revelaram a percepção dos participantes de que estavam não apenas revisando um instrumento, mas participando de um processo de valorização profissional, troca de saberes e reconstrução do cuidado. Destacaram o sentimento de pertencimento, a importância do espaço de escuta e o reconhecimento de suas experiências como fundamentais para a construção de um cuidado mais qualificado.

O Segundo Encontro retomou os principais pontos discutidos anteriormente e avançou na análise do instrumento. Por meio de discussões aprofundadas, foram propostas mudanças significativas, incluindo a adição do item 55, os demais itens foram reformulados em sua redação, considerando as necessidades apontadas pelos participantes.

Além do Quadro 1, há o material com os descritores de cada item do instrumento de Estratificação de Risco. Esses descritores também foram avaliados pelos participantes e sofreram modificações.

**Quadro 1:** Instrumento de Estratificação de Risco em Saúde Mental adequado enviado para avaliação dos juízes

ESTRATIFICAÇÃO DE RISCO EM SAÚDE MENTAL						
**Nome:**		Nº de Prontuário:				
DN:		Idade:				
Orientação Sexual:		Escolaridade:				
Ocupação:		Raça/cor:				
Estado civil:						
Nome e inscrição profissional:						
Serviço de saúde:			Data:			
**SINAIS E SINTOMAS**					**NÃO**	**SIM**
GRUPO I (Sintomas relacionados aos transtornos mentais comuns ou menores)	1. Ansiedade com ou sem sensação de pânico				0	4
	2. Insônia ou hipersônia				0	3
	3. Fobia (medo intenso de algo sem risco real)				0	2
	4. Crises conversivas				0	2
	5. Crises dissociativas				0	2
	**6.** Alterações do apetite **e do comportamento alimentar**				0	2
	7. Preocupação excessiva com o peso ou a forma corporal				0	2
	**8. Sintomas hipocondríacos** e/ou queixas físicas **sem causa orgânica**				0	2
	**9. Pensamentos obsessivos e/ou comportamentos compulsivos**				0	2
	10. Pensamentos de inutilidade e/ou sentimento de culpa				0	4
	11. Tristeza persistente com perda de interesse e prazer e/ou desesperança				0	4
	12. Apatia com ou sem isolamento social				0	4
	13. Prejuízo da atividade sexual				0	2
	14. Desorientação temporal e/ou espacial				0	2
GRUPO II (Sintomas relacionados aos transtornos mentais severos e persistentes)	**15. Pensamentos passivos de morte**				0	2
	**16. Ideação suicida sem planejamento**				0	4
	**17. Ideação suicida com planejamento ou recente tentativa de suicídio.**				0	10
	18. Humor instável com impulsividade ou destrutividade				0	6
	19. Heteroagressividade ou autoagressividade				0	8
	20. Desinibição social, sexual ou perda de pudor.				0	4
	21. Hiperatividade motora				0	4
	22. Humor elevado, expansivo, irritável ou eufórico				0	4
	23. Delírio				0	8
	24. Alucinação				0	8
	25. Alteração do curso e/ou da forma do pensamento				0	6
	26. Perda da capacidade crítica da realidade				0	8
	27. Alteração da memória				0	2
GRUPO III (Sintomas relacionados à dependência de álcool e outras drogas)	*28. Delirium tremen**s***				0	10
	29. Sinais ou sintomas de abstinência ao uso continuado de álcool e/ou drogas				0	8
	30. Incapacidade de redução e controle do uso de drogas				0	8
	31. Comportamento de risco, para si ou terceiros, sob efeito de álcool ou drogas				0	8
	32. Tolerância ao efeito do consumo de álcool ou drogas **lícitas e ilícitas**				0	6
	33. Uso **nocivo** de álcool ou drogas				0	8
GRUPO IV (Sintomas relacionados a alterações na saúde mental que se manifestam na infância e/ou na adolescência)	34. Dificuldade de compreender e/ou transmitir informação verbal manifesta no período de desenvolvimento infantil				0	4
	35. Movimentos corporais ou comportamentais repetitivos**, atípicos** ou paralisados.				0	4
	36. Dificuldade para adquirir e desenvolver as habilidades escolares				0	4
	37. Dificuldade para adquirir e desenvolver as habilidades motoras				0	4
	38. Severa dificuldade na interação social e às mudanças na rotina				0	8
	39. Desatenção **e/ou hiperatividade** c om interrupção prematura de tarefas e/ou deixando tarefas inacabadas				0	2
	40. Comportamento provocativo, desafiador e/ou opositor persistente				0	6
	41. Comportamentos ou reações emocionais que não correspondem ao esperado para a idade biológica				0	4
GRUPO V (Fatores que podem se constituir em agravantes ou atenuantes de problemas de saúde mental já identificado, incluindo condições especiais e eventos agudos)	42. Resistência, refratariedade ou não adesão ao tratamento.				0	4
	43. Recorrência ou **Recidiva** (após 2 meses **de estabilização)**				0	4
	44. Exposição continuada ao estresse ou evento traumático				0	4
	45. Precariedade de suporte familiar e/ou social				0	4
	46. Testemunha de violência				0	2
	47. Autor ou vítima de violência interpessoal				0	6
	48. Perda da autonomia				0	6
	49. **Perda da capacidade funcional**/ocupacional decorrente de agravo de saúde				0	4
	50.Vunerabilidade Social				0	2
	51. Histórico familiar de transtorno mental/ dependência química / suicídio				0	2
	52. Comorbidade ou outra condição crônica de saúde				0	4
	53. Faixa etária < 18 anos e > de 60 anos				0	2
	54. Abandono e/ou atraso escolar				0	2
	**55.Polifarmácia**				0	2
PONTUAÇÃO	RISCO					
0 a 42	BAIXO RISCO					
43 a 71	MÉDIO RISCO					
72 a 244	ALTO RISCO					
PONTUAÇÃO TOTAL:						

Durante a plenária, os dois grupos apresentaram as sugestões de adequações, debateram os pontos de divergência e buscaram construir consensos. Todas as modificações e inclusões estão sistematizadas no Quadro 1 (instrumento readequado), com os destaques em negrito.

Essa abordagem permitiu o alinhamento dos diferentes saberes trazidos pelos participantes em torno de uma temática comum, promovendo uma compreensão mais aprofundada e favorecendo a reflexão sobre a prática.

Para avaliação do segundo encontro as seguintes perguntas: “Como me sinto tendo participado dessa construção?”, “Quais contribuições da oficina levo para o trabalho?”, “Qual a expectativa com o instrumento de estratificação de risco para minha prática profissional?”.

As principais palavras mencionadas na avaliação final do Segundo Encontro, foram: oportunidade, troca experiência, capacitação, qualificar, auxílio, olhar integral e acolhimento, que sintetizam o impacto formativo da atividade, tanto no aprimoramento técnico do instrumento quanto no fortalecimento dos vínculos entre os profissionais e suas práticas cotidianas. Fato que favorece o aperfeiçoamento do processo de trabalho desses profissionais de saúde.

Ao longo dos encontros, observou-se o crescimento do grupo em relação à apropriação do processo e ao compromisso com a qualificação do cuidado em saúde mental. A escuta mútua, o respeito às diferentes experiências e o desejo coletivo de construir uma ferramenta mais sensível à realidade local contribuíram para que a oficina se configurasse como uma experiência significativa de educação permanente em saúde.

Em síntese, o processo resultou não apenas na construção de um instrumento adequado à realidade do município, mas também na qualificação do processo de trabalho das equipes de saúde, contribuindo, consequentemente, para a melhoria do cuidado às pessoas em sofrimento mental.

A escolha pela pedagogia dialógica de Paulo Freire como base para a organização da oficina evidenciou o valor das práticas participativas como estratégia para fortalecer vínculos profissionais, promover a corresponsabilização e qualificar a atenção em saúde mental.

## DISCUSSÃO

A oficina organizada a partir da pedagogia dialógica de Paulo Freire possibilitou a adequação de um Instrumento de Estratificação de Risco em Saúde Mental considerando a realidade de um município de médio porte. Essa vivência colaborativa entre os profissionais da RAPS que participaram da oficina favoreceu a identificação de lacunas no instrumento original e permitiu a inserção e/ou alteração de critérios mais claros ao cotidiano dos serviços e às especificidades da população atendida, conforme orienta a literatura sobre tecnologias avaliativas em saúde^(6,7)^.

Mais que uma estratégia participativa, a oficina é essencial para estimular a troca de saberes, capacitar e impulsionar a melhoria contínua dos profissionais, qualificando o cuidado em saúde mental. Ela favorece uma abordagem colaborativa, promovendo aprendizado, troca de experiências e construção de habilidades entre profissionais de diferentes áreas. Além disso, cria um ambiente para compartilhar vivências, discutir casos e analisar criticamente o fluxo de atendimento, ajustando os critérios de risco às realidades observadas^(8)^.

Um dos principais resultados observados foi a ressignificação do Instrumento de Estratificação de Risco em Saúde Mental pelos próprios participantes do estudo. Ao analisarem os critérios inicialmente propostos, os participantes puderam propor ajustes e criar novas categorias, a partir da sua experiência cotidiana. Esse movimento dialoga com a ideia de que instrumentos técnicos devem ser contextualizados, legitimados pelo coletivo e capazes de refletir a realidade de quem os aplica^(9)^.

A diversidade profissional do grupo, embora tenha imposto desafios quanto ao alinhamento de percepções, também enriqueceu o processo, pois possibilitou múltiplos olhares sobre o risco em saúde mental. A literatura tem enfatizado que a construção interprofissional de ferramentas avaliativas amplia sua legitimidade e potencializa o uso no cotidiano^(10)^.

Além disso, a construção participativa do instrumento contribuiu para a apropriação de sua finalidade pelos profissionais, o que tende a favorecer sua aplicação prática e melhoria do processo de trabalho das equipes de saúde. A compreensão do “porquê” e “para que” utilizar um instrumento é fator determinante para sua efetividade nos serviços, conforme indicam estudos sobre implementação de tecnologias em saúde^(5,7)^.

A estratégia adotada permitiu uma reflexão sobre o fluxo do cuidado em saúde mental na RAPS, promovendo análise crítica das rotinas e incentivando práticas inovadoras. O pertencimento gerado nos encontros fortaleceu o compromisso dos participantes com a adequação do instrumento, demonstrando que processos participativos são essenciais para a criação de tecnologias mais resolutivas, validadas contextualmente e orientadas à integralidade do cuidado^(9,10)^.

Por fim, a participação ativa dos profissionais da RAPS em um processo dialógico e democrático para a adequação do Instrumento de Estratificação de Risco em Saúde Mental é uma estratégia com potencial para qualificar o cuidado em saúde mental na APS, de forma crítica e contextualizada, promovendo melhorias no processo de trabalho das equipes de saúde.

### Limitações do estudo

O estudo enfrentou limitações como a baixa adesão dos gestores municipais à adequação do instrumento de Estratificação de Risco em Saúde Mental, o que poderá favorecer sua futura implantação. Além disso, a abordagem participativa adotada, possibilitando a intensa troca subjetiva entre os participantes, pode não ter captado plenamente as expressões e percepções coletivas, apesar dos dados obtidos terem sido suficientes para sustentar as análises e refletir a experiência vivenciada.

### Contribuições para a Área

Ao adotar a pedagogia freireana, o estudo promove uma abordagem emancipadora, fortalecendo práticas críticas na saúde e qualificando o processo de trabalho das equipes, em sintonia com os princípios do SUS. Contribui para a Enfermagem ao propor melhorias no cuidado, incentivar a corresponsabilidade em saúde mental e valorizar o protagonismo das equipes na criação de tecnologias adequadas aos territórios.

## CONSIDERAÇÕES FINAIS

Este relato de experiência evidenciou a potência da oficina como espaço de formação, escuta e reconstrução coletiva do cuidado em saúde mental na APS. A adequação do Instrumento de Estratificação de Risco em Saúde Mental, por meio da participação ativa de profissionais da RAPS, mostrou-se não apenas um exercício técnico de revisão de itens, mas uma prática pedagógica viva, que conectou saberes, realidades e afetos.

As contribuições dos participantes de diferentes categorias profissionais melhoraram os critérios de estratificação e ampliaram a compreensão sobre o sofrimento psíquico. Ao final do processo, os participantes não apenas modificaram o instrumento, mas se reconheceram como sujeitos ativos na construção de um cuidado mais qualificado, humano e territorializado.

A revisão do instrumento, com base na experiência prática, refinou os critérios e aumentou sua clareza, fortalecendo seu papel no apoio à decisão clínica, à organização do cuidado em saúde mental e, consequentemente, na melhoria do processo de trabalho das equipes de saúde.

## Data Availability

Os dados de pesquisa só estão disponíveis mediante solicitação.
